# A comprehensive image dataset of jute diseases

**DOI:** 10.1016/j.dib.2025.112334

**Published:** 2025-11-28

**Authors:** Md. Masudul Islam, Md. Ripon Sheikh

**Affiliations:** Bangladesh University of Business and Technology, Dhaka, Bangladesh

**Keywords:** Jute dataset, Jute disease, Agriculture, Crop disease, Computer Vision, Image Dataset

## Abstract

This Data Descriptor presents the Jute Diseases Image Dataset; a curated collection of 1390 high-resolution images aimed at supporting the development of machine learning models for timely identification and accurate diagnosis of jute (Corchorus) plant diseases. The dataset is categorized into five classes: Dieback (300), Holed (300), Mosaic (240), Stem Soft Rot (270), and Fresh (280) representing healthy leaves. Images were captured under varied natural lighting and directional conditions across diverse jute cultivation areas to enhance model generalizability. A rigorous pre-processing pipeline was applied, including uniform resizing to 1024 × 1024 pixels and removal of duplicate images to ensure data integrity. The dataset is organized into two components: a raw, pre-processed set and an augmented train-test split version, enabling immediate use in machine learning workflows. Additionally, Grad-CAM and Guided Grad-CAM techniques were applied to sample images to visualize and validate model attention on disease-relevant regions. This resource addresses the lack of labelled jute disease imagery and supports timely disease management, particularly for stakeholders in Bangladesh and other major jute-producing regions.

Specifications Table

**Table:** 

Subject	Computer Sciences
Specific subject area	Computer Vision, Pattern Recognition, machine learning, deep learning, Agriculture
Type of data	JPG Raw Image
Data collection	The dataset comprises 1390 high-resolution images of jute plants captured using smartphone cameras under varied natural lighting and directional conditions across key jute-growing regions in Bangladesh (Faridpur, Rajbari, Magura, Jamalpur, Kushtia, Dhaka, Mymensingh, Comilla) from May to June period. Images were categorized into five classes: Dieback, Holed, Mosaic, Stem Soft Rot, and Fresh (healthy). Data pre-processing included uniform resizing to 1024 × 1024 pixels (JPG format), duplicate removal, and expert validation. Augmentation (rotations, flips, brightness/contrast adjustments) was applied using image processing tools. The dataset is split into 80 % training and 20 % testing sets, maintaining class balance for computer vision research purpose.
Data source location	The images were collected across key jute-growing regions in Bangladesh, including Faridpur, Rajbari, Magura, Jamalpur, Kushtia, Dhaka, Mymensingh, and Comilla. The dataset is stored in Harvard Dataverse and was curated by researchers from the Bangladesh University of Business and Technology, Dhaka
Data accessibility	Repository name: **Harvard Dataverse** Data identification number: https://doi.org/10.7910/DVN/FJ1DM1 Direct URL to data: https://dataverse.harvard.edu/dataset.xhtml?persistentId=doi:10.7910/DVN/FJ1DM1 Instruction for accessing these data: **Go to the download link and simply go to the ‘File’ tab and click download icon for download the data or click ‘Eye’ icon to view the dataset**.
Related research article	

## 1 Value of the Data

• This dataset provides high-quality, labeled images of jute diseases, addressing a critical gap in agricultural AI research. It enables the development of machine learning models for early disease detection, benefiting crop management and food security in jute-dependent regions like Bangladesh.
• Jute is a major cash crop, and diseases cause significant yield losses. This dataset supports AI-driven tools for farmers, helping them identify diseases early and apply timely interventions, reducing economic losses and improving productivity.
• Researchers can reuse this dataset to train, test, and benchmark computer vision models for plant disease detection, transfer learning, or comparative studies in agricultural AI. Although the symptoms are visible, the dataset enables scalable digital classification, documentation, and comparative analysis—useful where expert access or consistent diagnosis is limited.
• Beyond agriculture, these data can be used in environmental studies, remote sensing, and educational tools, fostering innovation in sustainable farming and digital agriculture technologies. Although most samples represent visible disease stages, the dataset supports timely and automated recognition of jute diseases under field conditions.
• The dataset also holds interdisciplinary potential, supporting applications in public policy planning (for crop disease management and monitoring), supply chain optimization (predicting raw jute quality for textile industries), and educational tools that promote digital agriculture awareness and sustainability.

## 2 Background

Jute is a vital crop for Bangladesh’s economy, but diseases like stem rot [1], mosaic [2], and dieback [3] cause severe yield losses. Stem rot and mosaic diseases alone can reduce jute yields by up to 25–40 % in major producing areas [12], highlighting the urgency of timely identification. Traditional visual diagnosis by farmers is often delayed, leading to poor disease management. While computer vision-based collection of datasets exists for animals, crops, fruits and vegetables [[4], [5], [6], [7], [8], [9], [10]], the lack of a standardized, labelled dataset for jute diseases hindered progress in this field. This dataset was created to fill that gap, providing high-resolution, expert-validated images of diseased and healthy jute plants. Images were captured under real-world field conditions to ensure ecological validity. Methodologically, the dataset follows best practices in agricultural AI, including rigorous pre-processing (resizing, deduplication) and augmentation (rotations, lighting variations) to enhance machine learning robustness. This state-of-the-art dataset supports the development of computer vision models for automated disease detection, aligning with global efforts to digitize agriculture. It also complements ongoing research in plant pathology and precision farming by offering a foundational resource for benchmarking and model training.

## 3 Data Description

**Fig. 1:**
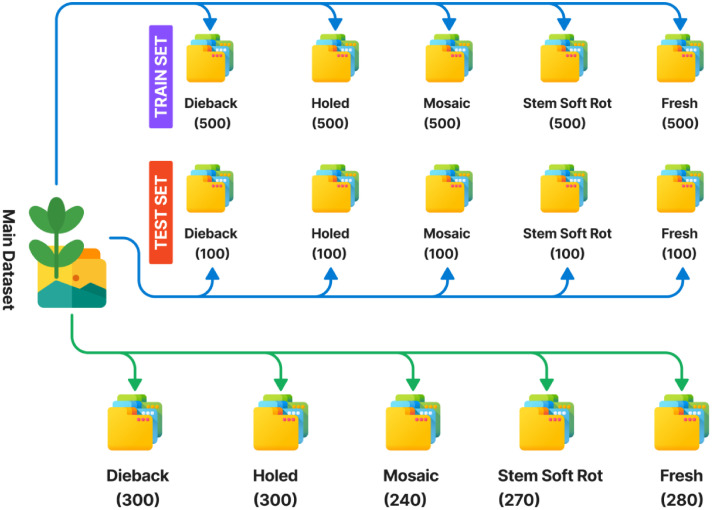
Dataset Organization Structure.

**Fig. 2:**
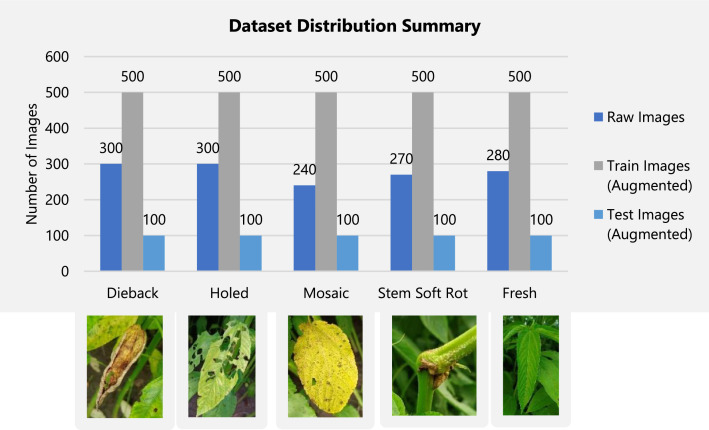
Example Image Data and Dataset Summary.

The dataset stored in Harvard Dataverse repository [11] is organized into two main folders: “Jute Disease Dataset” and “Jute Disease Dataset-ML”, each containing structured subfolders for easy navigation. The “Jute Disease Dataset” folder houses five subfolders—”Dieback”, “Holed”, “Mosaic”, “Stem_Soft_Rot”, and “Fresh”—each containing original, preprocessed 1024 × 1024-pixel JPEG images of jute leaves, with 300, 300, 240, 270, and 280 images per class, respectively. The “Jute Disease Dataset-ML” folder includes two subfolders: “TRAIN” and “TEST”, which follow the same class-wise structure but contain augmented images (e.g., rotated-90 to 180 degrees, flipped, brightness-adjusted) resized to 512 × 512 pixels. The “TRAIN” subfolder holds 80 % of the data (2500 images), while “TEST” contains the remaining 20 % (500 images), maintaining proportional class distribution. The pre-augmented set is provided as an optional ready-to-use version for rapid benchmarking and educational purposes, while the raw images remain available for customized augmentation workflows. In Fig. 1 it illustrates the folder structure of our dataset organization, and Fig. 2 summarizes the image counts per class. Table 1 represents the raw and augmented images summary.

**Table 1:** Summary of Image Counts for Raw and Augmented Datasets with example images.

Class Name		Raw Images	Augmented Images (train)	Unique Image (Test)
Dieback		300	500	100
Holed		300	500	100
Mosaic		240	500	100
Stem Soft Rot		270	500	100
Fresh (Healthy)		280	500	100
Total		**1390**	**2500**	**500**

## 4 Experimental Design, Materials and Methods

**Fig. 3:**
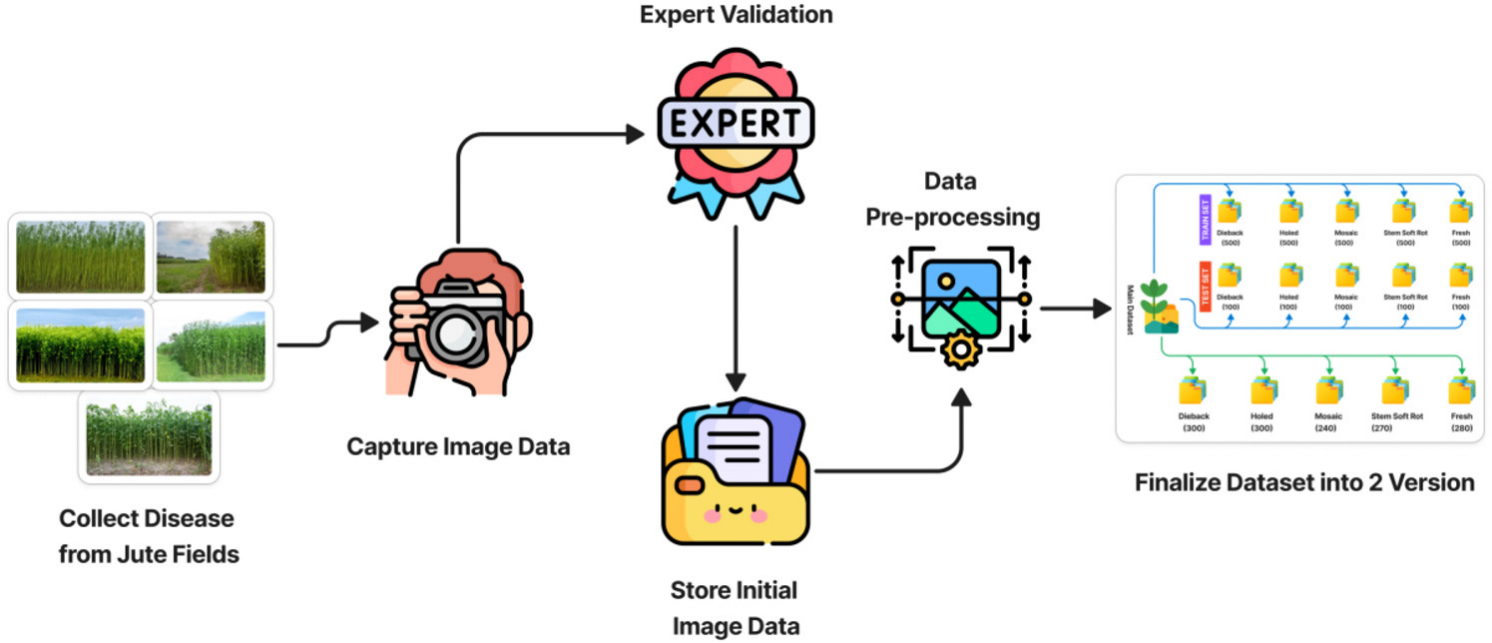
Dataset Collection Process step by step.

The dataset was acquired through a systematic field-based imaging campaign conducted during the peak jute cultivation season (March-July) across 8 major jute-growing regions in Bangladesh: Faridpur, Rajbari, Magura, Jamalpur, Kushtia, Dhaka, Mymensingh, and Comilla. Image collection was performed using iPhone 11 smartphone cameras with resolutions ranging from 48 Mega Pixel, capturing both close-up leaf images (20–30 cm distance) and whole-plant shots (1–1.5 m distance). All images were taken under natural field conditions with varying lighting (direct sunlight, overcast, partial shade) and angles (top-down, side, oblique) to ensure ecological validity. All disease categories were validated by two agricultural experts specializing in jute farming. Each image was independently cross-checked, and ambiguous cases were jointly reviewed or excluded to ensure labeling accuracy and consistency across classes. Field experts provided real-time guidance via mobile consultations to verify disease symptoms and ensure accurate visual classification into five categories: Dieback (caused by Diplodia corchori), Holed (general physical damage), Mosaic (viral), Stem Soft Rot (Macrophomina phaseolina), and Fresh (healthy). The raw dataset of 1390 images was initially preprocessed using ACDSee Photo Studio Ultimate, where images were batch-renamed, resized to 1024 × 1024 pixels, and manually inspected for duplicates. Augmentation was performed using the same software, applying random transformations including rotation (±90°), horizontal/vertical flips, brightness adjustment (±30 %), contrast variation (±20 %), and minor color jittering (±5 % saturation/hue). The machine learning suitable version of our dataset is resized to 512 × 512 pixel resolution. The augmented dataset was then split into training (80 %) and testing (20 %) sets. To ensure reproducibility, this split was performed using a fixed random seed value (42), preserving the same data partitioning across multiple experiments. In Fig. 3 the work-flow of our image data acquisition process are shown in step by step.

**Fig. 4:**
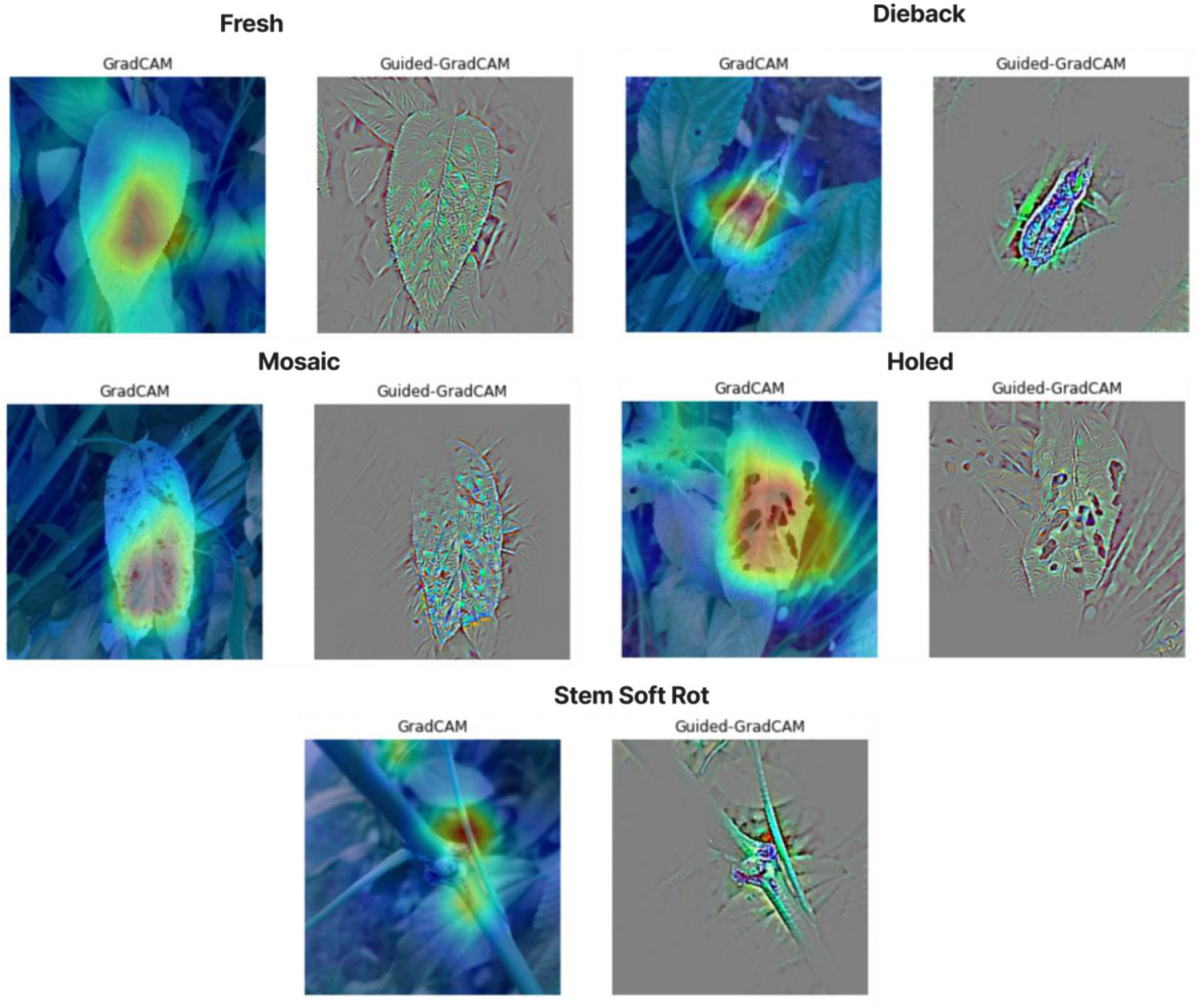
GradCAM and Guided GradCAM of Example Images.

Grad-CAM and Guided Grad-CAM were included in this study to enhance the interpretability of machine learning models for jute disease detection. Grad-CAM visualizations were generated using a fine-tuned MobileNetV2 classifier trained on the augmented dataset (accuracy 92.4 %, last convolutional layer ‘Conv_1′). These were used solely for interpretability demonstration. These visualization techniques highlight the regions in leaf images that most influence the model's predictions, ensuring decisions are based on biologically relevant features (e.g., lesions, discoloration) rather than artifacts. By validating attention maps against expert annotations, we confirm model reliability for real-world agricultural use. This transparency bridges the gap between AI outputs and actionable farmer insights, fostering trust in automated diagnostics while advancing explainable AI in precision agriculture. Fig. 4 shows the Grad-CAM and Guided Grad-CAM interpretation of example image from our dataset.

### 4.1 Camera specification

All the images were captured using an iPhone 11 equipped with a dual-camera system, consisting of a 12MP Wide lens (ƒ/1.8 aperture, 26 mm focal length) and a 12MP Ultra-Wide lens (ƒ/2.4 aperture, 120° field of view). The setup supported 2x optical zoom and 5x digital zoom. Capture settings included studio lighting, 9MP resolution, ISO 125–135, and a fixed distance of approximately 4.2 inches from subjects.

## Limitations

The dataset has several limitations. First, image collection was restricted to specific regions in Bangladesh, potentially limiting geographic diversity and generalizability to other jute-growing areas. Second, while efforts were made to capture varied lighting conditions, reliance on natural light introduced inconsistencies in brightness and contrast across images. Third, the dataset's size (1390 images) may be insufficient for training highly complex deep learning models without augmentation. Fourth, the “Holed” class encompasses diverse damage causes (e.g., insects, abiotic factors) without pathogen-specific differentiation, which could reduce diagnostic precision. Fifth, smartphone cameras, despite their resolution, lack the controlled conditions of specialized imaging systems, potentially affecting fine-detail capture. Also, the dataset primarily captures visible disease symptoms; future work should include time-series images for early-stage prediction. These limitations highlight opportunities for future expansions in geographic coverage, imaging standardization, and pathological specificity.

## Ethics Statement

This article does not involve any research involving human or animal subjects by any of the authors. The datasets utilized in this article are publicly accessible. When utilizing these datasets, it is essential to adhere to appropriate citation guidelines.

## Credit Author Statement

**Md. Masudul Islam:** Software, Validation, Formal Analysis, Methodology, Writing - Original Draft, Visualization. **Md Ripon Sheikh:** Conceptualization, Resources, Writing - Original Draft, Data Curation.

## Data Availability

DataverseJute Disease Image Dataset (Original data).

## References

[1] Mandal K., Datta S., De R., Sarkar S. (Apr. 2025). Jute under siege: a deep dive into stem rot disease caused by Macrophomina phaseolina (Tassi) Goid. Crop Protect..

[2] Mahmud (Jan. 20, 2014). Management of jute yellow mosaic virus disease through cultural practices. Arch. Phytopathol. Plant Protect..

[3] Pamala P.J., Jayalakshmi R.S., Vemana K., Naidu G.M., Varshney R.K., Sudini H.K. (Dec. 04, 2023). Prevalence of groundnut dry root rot (Macrophomina phaseolina (Tassi) Goid.) And its pathogenic variability in Southern India. Front. Fungal Biol..

[4] Himel G.M.S., Islam M.M. (Feb. 2024). GalliformeSpectra: a hen breed dataset. Data Brief.

[5] Bai L., Zhang Z., Song J. (Oct. 2024). Image dataset for cattle biometric detection and analysis. Data Brief.

[6] Islam M.M., Himel G.M.S., Uddin M.S., Moazzam M.G. (Jun. 2024). A visual dataset for recognition of rice varieties. Data Brief.

[7] Tahsin M. (Jun. 2025). PaddyVarietyBD: classifying paddy variations of Bangladesh with a novel image dataset. Data Brief.

[8] Sheikh M.R., Islam M.M., Himel G.M.S. (Apr. 2024). LuffaFolio: a multidimensional image dataset of smooth Luffa. Data Brief.

[9] Ahmed M.J., Saha R., Dutta A.K., Mojumdar M.U., Chakraborty N.R. (Apr. 2025). BanglaVeg: a curated vegetable image dataset from Bangladesh for precision agriculture. Data Brief.

[10] Bharati R.K., Islam M.M., Sheikh M.R., Himel G.M.S. (Jun. 2025). A comprehensive image dataset of Bangladeshi mango variety. Data Brief.

[11] Islam M.M., Sheikh M.R. (2025). Jute disease image dataset. Harvard Dataverse.

[12] Mandal K., Datta S., De R.K., Sarkar S.K. (Jan. 2025). Jute under siege: a deep dive into stem rot disease caused by Macrophomina phaseolina (Tassi) Goid. Crop Protect..

